# The route of SARS-CoV-2 to brain infection: have we been barking up the wrong tree?

**DOI:** 10.1186/s13024-022-00529-9

**Published:** 2022-03-15

**Authors:** Rafal Butowt, Christopher S. von Bartheld

**Affiliations:** 1 L. Rydygier Collegium Medicum, Nicolaus Copernicus University, 85-094 Bydgoszcz, Poland; 2 Department of Physiology and Cell Biology, University of Nevada, Reno School of Medicine, Reno, NV 89557-0352 USA

**Keywords:** COVID-19, SARS-CoV-2, Brain infection, ACE2, Olfactory system, Nervus terminalis, Omicron, TMPRSS2

## Abstract

This letter draws attention to recent work supporting the notion that the SARS-CoV-2 virus may use the nervus terminalis rather than the olfactory nerve as a shortcut route from the nasal cavity to infect the brain.

## To the editor

### Main text

It is well-established that, in rare cases, proteins and/or RNA of SARS-CoV-2 (the virus causing the COVID pandemic) can be found in the brain of patients suffering from COVID-19 [1, 2]. It is also well-established that replicating SARS-CoV-2 can infect the brain in some animal models [3, 4], although such evidence is still lacking for the human brain. Initially, it appeared reasonable to assume that SARS-CoV-2 travels along the olfactory nerve from the nose to the brain, for several reasons: The highest viral load was reported in the olfactory epithelium [1], other viruses are known to be neurotropic and can invade the brain via the olfactory nerve [3, 5, 6], and most COVID-19 patients infected with the virus variants dominating in 2020-2021 experienced loss of smell [3].

But does SARS-CoV-2 use the olfactory nerve as a portal to brain infection (or brain exposure) as assumed by many authors [1, 4]? Critical examination of the evidence revealed a number of inconsistencies with this notion:

1. The obligatory viral entry protein angiotensin-converting enzyme 2 (ACE2) is not expressed in olfactory receptor neurons, raising doubts that SARS-CoV-2 can enter these neurons and travel along their axons into the brain [3, 7].
2. SARS-CoV-2 infects olfactory receptor neurons not at all or very rarely [1, 3, 8].
3. SARS-CoV-2 and shed virus proteins such as subunit S1 can reach the brain without accumulating in the olfactory bulb [3, 9, 10].
4. The time course of SARS-CoV-2 brain infection/exposure is incompatible with a neuron-hopping scenario in animal models, further raising doubt about the notion that SARS-CoV-2 is a true neurotropic virus as shown for several other viruses [3, 6].

If SARS-CoV-2 does not travel along the olfactory nerve, how can it reach the brain? Importantly, there is another cranial nerve that runs close to the olfactory nerve into the brain: the nervus terminalis. This little-known cranial nerve connects the olfactory epithelium directly with brain structures caudal to the olfactory bulb. It exists in humans where it contains several hundred to more than one thousand neurons [11, 12]. Three new studies shed more light on this alternate route.

- Bilinska et al. [12] showed that a major fraction of the neurons in the nervus terminalis express the virus entry protein ACE2. Since this cranial nerve connects the olfactory epithelium directly with the hypothalamus, the authors concluded that the nervus terminalis may provide a route for the virus to reach the brain. The variability in neuro-invasion seen in animal models, even within the same species, fits better with the known variability of the nervus terminalis system (which differs an order of magnitude between members of the same species) than with the uniform olfactory system [12].
- Sauve et al., in a not yet peer-reviewed preprint [9], showed that SARS-CoV-2 can skip the olfactory bulb on its way to the brain, but the virus does appear – in an animal model and in COVID-19 patients – in significant amounts in the hypothalamus, especially in the olfactory placode-derived neurons of the hypothalamus which also express the virus entry protein ACE2. This supports a previous report showing that shed spike protein (subunit 1) of SARS-CoV-2 appears more consistently in the hypothalamus than the olfactory bulb after nasal inoculation [10].
- By examining rapid biopsies of the olfactory epithelium from COVID-19 patients, Khan et al. [8] showed that the virus did not infect olfactory receptor neurons in humans, only non-neuronal cells, primarily sustentacular cells. The SARS-CoV-2 virus did not reach the olfactory bulb in COVID patients, even in those with anosmia. Anosmia in these patients appears to be caused by loss of support cell function, not by infection and loss of the olfactory receptor neurons or loss of neurons in the olfactory bulb [3, 7, 8].

**Fig. 1:**
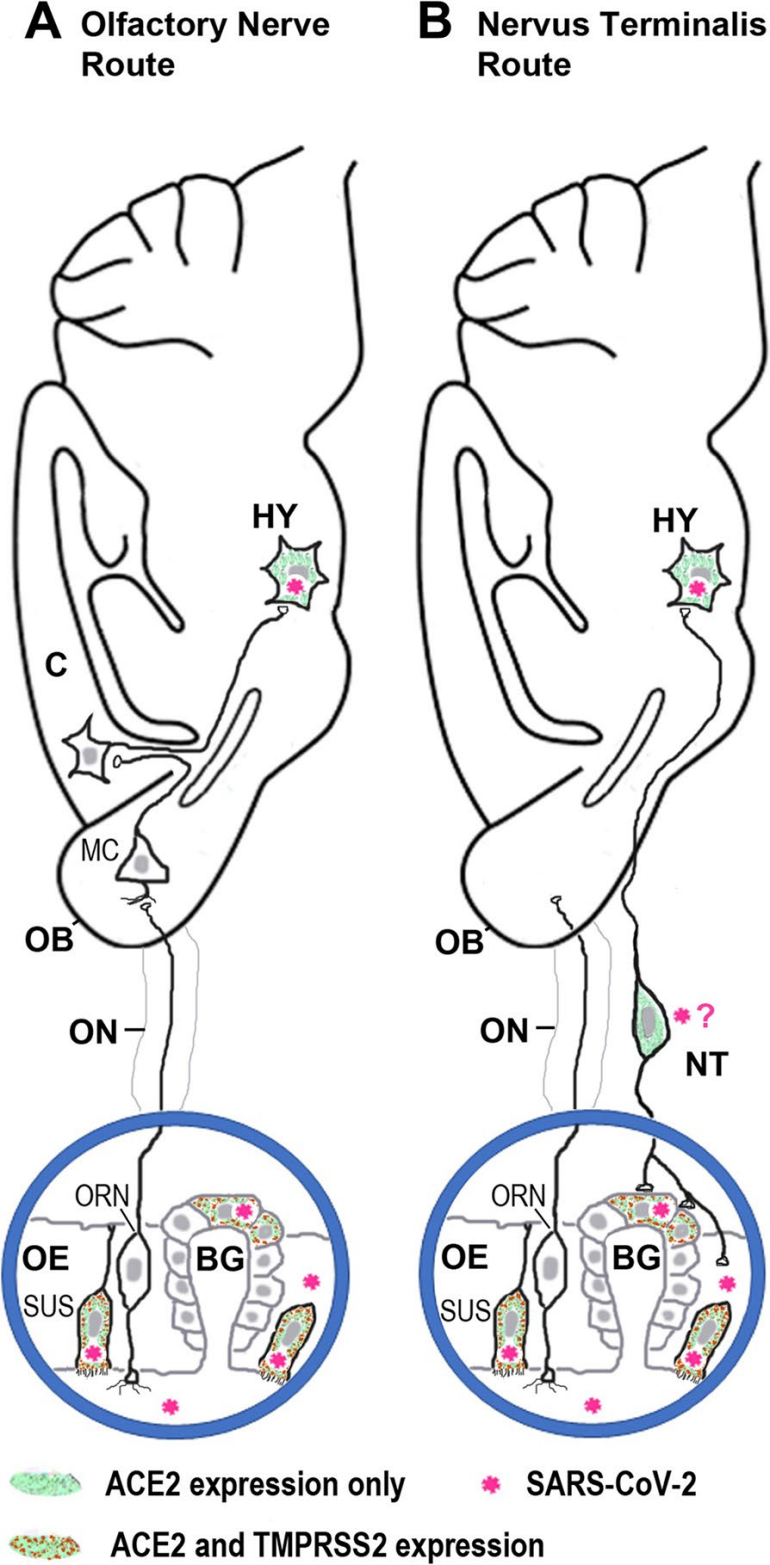
Schematic illustration of two routes how SARS-CoV-2 or the virus’ cleaved S1 subunit of the spike protein may travel from the nose to the brain. **A**. Route along the olfactory nerve (ON). Olfactory receptor neurons (ORNs) and most mitral cells (MCs) in the olfactory bulb (OB) do not express the obligatory viral entry protein, ACE2 (angiotensin-converting enzyme 2), are rarely or not at all infected by the virus, and the ON and the OB are not always infected when SARS-CoV-2 is found in the brain [3, 9]. Support cells in the olfactory epithelium express ACE2 and the surface protease TMPRSS2, and these cells (sustentacular cells, SUS) become infected with SARS-CoV-2 [3, 7, 8]. C, cerebral cortex; Hy, hypothalamus. **B**. Route along the nervus terminalis (NT). This cranial nerve connects the olfactory epithelium, and in particular Bowman gland (BG) cells, directly with nuclei beyond the olfactory bulb (OB), including the hypothalamus [11, 12]. Support cells (SUS) and BG cells express ACE2 and TMPRSS2 and are known to become infected by SARS-CoV-2 [3, 7, 8]. Nervus terminalis (NT) neurons also express ACE2, as do neurons in the hypothalamus (HY) which become infected by SARS-CoV-2 [9]. NT neurons and endocrine neurons in the hypothalamus do not express TMPRSS2, but they express neuropilin 1 [9] or cathepsins [12], other proteases that can mediate virus membrane fusion. Whether NT neurons become infected by SARS-CoV-2 or by its cleaved S1 spike protein remains to be determined

Taken together, these three studies support the notion that SARS-CoV-2 (or its shed spike protein) does not utilize the olfactory nerve to reach the brain, but rather uses a shortcut: neurons of the nervus terminalis (the little-known cranial nerve “0” [12]) which innervates the nasal cavity, and within the olfactory epithelium specifically the ACE2-expressing (and virus-accumulating) cells in Bowman glands [7] (Fig. 1). The nervus terminalis neurons have central processes that project directly to targets in the brain including the hypothalamus, bypassing the olfactory bulb. Once the hypothalamus is reached, SARS-CoV-2 can penetrate the blood-brain-barrier and can reach various neural circuits connected to the hypothalamus, including brainstem nuclei that are involved in respiration. Interestingly, the hypothalamus and also the choroid plexus express ACE2 [13], and this may provide a basis for dysfunction of the renin-angiotensin system (RAS) and blood pressure dysregulation, especially in elderly COVID patients who have a compromised RAS due to aging and who are particularly at risk for severe COVID [13].

Alternatively, SARS-CoV-2 may infect nervus terminalis neurons that connect the olfactory epithelium with spaces containing cerebrospinal fluid (CSF) [5, 12] (Fig. 1). Such access would explain the rapid spread (within less than 24 h after nasal inoculation) of SARS-CoV-2 to the CSF that has been described in some animal models [4] – a time course that is incompatible with a neuron-hopping route (which takes several days) as is known for other neurotropic viruses [3, 5, 6]. Regardless whether whole SARS-CoV-2 (or its spike protein or RNA) moves from neuron to neuron, or disseminates via CSF spaces [5], or by hematogenous spread – the nervus terminalis may facilitate all three of these modes of dissemination from the nose due to its unique anatomical connections [11, 12]. Hematogenous spread with breach of the blood brain barrier appears to be a less likely route, because in most studies of patients with COVID-induced neurological symptoms, evidence for SARS-CoV-2 RNA was not found in the patient’s CSF [3].

Is exposure to the S1 subunit of the spike protein sufficient to induce neuroinflammation in the brain? Recent work on the significance of the S1 subunit – the protein which contains the ACE2 receptor binding domain – has shown two important concepts. First, as already mentioned, Rhea et al. [10] established that the cleaved S1 subunit can be transported from the olfactory epithelium to the hypothalamus, apparently by binding to ACE2. Second, Frank et al. [14] showed that the S1 subunit, but not the S2 subunit, is sufficient to elicit neuroinflammation, including microglia activation and gene expression of multiple pro-inflammatory cytokines, as well as altered animal behavior reminiscent of neurological and cognitive symptoms in COVID-19 patients. Since microglia do not express ACE2 [14], these effects are likely mediated by binding of the S1 protein to pattern recognition receptors such as those of the toll-like receptors, especially TLR4 [14]. Accordingly, emerging evidence suggests that the cleaved S1 subunit travels along the ACE2-expressing nervus terminalis neurons and their axons into the brain, and the spike protein subunit 1 may then activate microglia by binding to TLR4, resulting in enhanced expression of pro-inflammatory cytokines such as IL1b and antigen-presenting molecules such as MHCII – molecules that are also upregulated in postmortem brains of COVID-19 patients. Thus, a full-blown infection of the brain with replicating SARS-CoV-2 is not necessary to induce neuroinflammation with neurological, cognitive, and neuropsychiatric symptoms that can be caused by microglia activation, synapse stripping and neuronal death [14].

The consequences of brain infection with, or exposure to, SARS-CoV-2 (or merely S1 spike protein) are still unclear. In general, the level of the virus and its proteins in the brain is very low, and there is no correlation between the extent of neurological symptoms and the extent of virus infection or exposure [2]. On one extreme, it has been proposed that when SARS-CoV-2 reaches brainstem respiratory centers, it can cause immediate death or may increase the risk for subsequent neurodegenerative diseases [2, 3]. On the other extreme, SARS-CoV-2 in the brain – if it does not replicate and does not induce inflammation – may have little negative consequences. Microglial activation could be responsible for the neurological and neuropsychiatric effects in COVID and enhance the risk of dementia and other neurodegenerative diseases, even when the microglia in the brain become activated not by infection with replicating SARS-CoV-2 virus, but rather by exposure to the virus’ spike protein [14].

Understanding the routes of brain infection has taken a new urgency with the emergence of the omicron variant. This variant replicates more abundantly in the upper airways as compared to lungs, leading to exceptional transmissibility, but it more efficiently uses endosomal host cell entry rather than the surface protease (TMPRSS2-mediated) route which means that it may be more infectious than previous variants in cells that do not express TMPRSS2 [15]. Since the nervus terminalis expresses ACE2 but not TMPRSS2 [12], similar to multiple cell types in the brain, this cranial nerve can be predicted to be a prime target and route for the omicron variant to reach the hypothalamus. In fact, this route may explain omicron’s increased symptoms of night sweats, nausea, and loss of appetite when compared with previous variants, since the hypothalamus is involved in the regulation of all three of these symptoms.

We now have a better grasp as to which tree we should be barking up and on which routes to focus for future experimental testing in animal models.

## Data Availability

All data can be found in the cited references which are publicly available.

## Abbreviations

ACE2: Angiotensin-converting enzyme 2
COVID-19: Coronavirus disease-19
CSF: Cerebrospinal fluid
IL1b: interleukin 1b
MHCII: Major histocompatibility complex class II
RAS: Renin-angiotensin system
SARS-CoV-2: Severe acute respiratory syndrome coronavirus 2
TLR4: Toll-like receptor 4
TMPRSS2: Transmembrane protease serine 2
